# Sleep Disruption Precedes Forebrain Synaptic Tau Burden and Contributes to Cognitive Decline in a Sex-Dependent Manner in the P301S Tau Transgenic Mouse Model

**DOI:** 10.1523/ENEURO.0004-24.2024

**Published:** 2024-06-14

**Authors:** Shenée C. Martin, Kathryn K. Joyce, Julia S. Lord, Kathryn M. Harper, Viktoriya D. Nikolova, Todd J. Cohen, Sheryl S. Moy, Graham H. Diering

**Affiliations:** ^1^Departments of Cell Biology and Physiology, Neuroscience Center, University of North Carolina at Chapel Hill, Chapel Hill, North Carolina 27599; ^2^Psychiatry, University of North Carolina at Chapel Hill, Chapel Hill, North Carolina 27599; ^3^Neurology, Neuroscience Center, University of North Carolina at Chapel Hill, Chapel Hill, North Carolina 27599; ^4^Carolina Institute for Developmental Disabilities, Carrboro, North Carolina 27510

**Keywords:** Alzheimer's disease, biological sex, sleep, sleep disruption, synapse, Tau

## Abstract

Sleep disruption and impaired synaptic processes are common features in neurodegenerative diseases, including Alzheimer's disease (AD). Hyperphosphorylated Tau is known to accumulate at neuronal synapses in AD, contributing to synapse dysfunction. However, it remains unclear how sleep disruption and synapse pathology interact to contribute to cognitive decline. Here, we examined sex-specific onset and consequences of sleep loss in AD/tauopathy model PS19 mice. Using a piezoelectric home-cage monitoring system, we showed PS19 mice exhibited early-onset and progressive hyperarousal, a selective dark-phase sleep disruption, apparent at 3 months in females and 6 months in males. Using the Morris water maze test, we report that chronic sleep disruption (CSD) accelerated the onset of decline of hippocampal spatial memory in PS19 males only. Hyperarousal occurs well in advance of robust forebrain synaptic Tau burden that becomes apparent at 6–9 months. To determine whether a causal link exists between sleep disruption and synaptic Tau hyperphosphorylation, we examined the correlation between sleep behavior and synaptic Tau, or exposed mice to acute or chronic sleep disruption at 6 months. While we confirm that sleep disruption is a driver of Tau hyperphosphorylation in neurons of the locus ceruleus, we were unable to show any causal link between sleep loss and Tau burden in forebrain synapses. Despite the finding that hyperarousal appears earlier in females, female cognition was resilient to the effects of sleep disruption. We conclude sleep disruption interacts with the synaptic Tau burden to accelerate the onset of cognitive decline with greater vulnerability in males.

## Significance Statement

Sleep disruption associated with Alzheimer's disease (AD) may be an important driver of disease pathology and cognitive decline. Current research suggests that sleep disruption worsens pathology, which further drives sleep disruption, forming a vicious feedback loop. Thus, it is crucial to understand the role of sleep disruption in AD. We tested the onset and consequences of sleep disruption in an AD mouse model. We find that sleep disruption is an early symptom of disease progression that accelerates cognitive decline. Notably, while females show an earlier onset of sleep disruption than males, females were comparably resilient and males comparably vulnerable to the negative effects of sleep disruption. Thus, sleep plays an important role in AD progression in a sex-specific manner.

## Introduction

Declines in sleep amount and quality are expected components of aging that contribute to susceptibility to develop Alzheimer's disease (AD; Bombois et al., 2010). During AD progression, sleep disruption tracks with cognitive decline and accumulation of neuropathology (Winer et al., 2019). Emerging work suggests a bidirectional relationship linking sleep disruption and AD pathology: sleep disruption promotes pathology, and pathology drives sleep disruption in a vicious feedforward cycle (Kang et al., 2017; C. Wang and Holtzman, 2019; Morrone et al., 2023). Promoting restorative sleep may be an important strategy to halt or delay pathology progression and protect cognition. Synapse dysfunction is known to occur in AD progression (Roberson et al., 2011). Synapses undergo widespread remodeling during sleep or in response to sleep disruption, suggesting synapses are a locus for the restorative benefits of sleep and site of dysfunction in response to sleep loss (de Vivo et al., 2017; Diering et al., 2017; Z. Wang et al., 2018; Bruning et al., 2019; Noya et al., 2019). Understanding the causes and consequences of sleep disruption and its links with synapse dysfunction is essential in developing effective sleep-based AD treatments.

Tau pathology is prevalent in AD and frontal temporal dementia (FTD). Tau is a microtubule-binding protein primarily localized to the neuronal axon (Kadavath et al., 2015; Prezel et al., 2018; Tapia-Rojas et al., 2019). During disease progression, phosphorylated Tau is relocalized to dendrites and postsynaptic compartments, where it contributes to synapse dysfunction and synapse loss (Ittner and Ittner, 2018). Sleep disruption promotes Tau aggregation and spread, whereas restorative sleep promotes Tau clearance (Holth et al., 2019). Recent studies examined sleep and the consequences of sleep disruption in the PS19 mouse model of AD/FTD that expresses an aggregation-prone human Tau P301S variant (Yoshiyama et al., 2007). PS19 AD/FTD mouse model exhibit Tau aggregation, neuroinflammation, synapse dysfunction, cognitive decline, and brain atrophy in an age-related manner that can be broadly described as presymptomatic at 3 months, early-stage disease at 6 months (sparse Tau pathology and minor changes in behavior), symptomatic disease stage at 9 months (widespread pathology and robust cognitive decline but without widespread neurodegeneration), and end-stage at 11–12 months (robust neuron loss, brain atrophy, and death; Yoshiyama et al., 2007; Dejanovic et al., 2018). Symptomatic 9–11-month-old PS19 males exhibit declines in rapid eye movement (REM) sleep and non-REM (NREM) sleep and reduced sleep bout duration (sleep fragmentation; Holth et al., 2017). Sleep amount negatively correlates with Tau pathology in sleep-promoting areas of the brainstem but not in the cortex, suggesting that Tau burden in sleep-promoting brain areas was a driver of age-related sleep disruption (Holth et al., 2017). Tau burden has also been observed in younger PS19 mice in the wake-promoting region of the brainstem, the noradrenergic locus ceruleus (LC; Zhu et al., 2018; Holth et al., 2019). Indeed, the LC is one of the first brain regions to show Tau pathology in humans and animal models, well in advance of many disease symptoms (Braak et al., 2011; Kelly et al., 2017). Chronic sleep disruption (CSD) in PS19 mice drove increased Tau burden in the LC, accelerating the loss of LC noradrenergic neurons (Zhu et al., 2018). Biological sex is also a critically important factor in understanding the role of sleep disruption in the progression of AD and Tau pathology. Males and females have been shown to have differential likelihoods of developing neurodegenerative conditions (Sinforiani et al., 2010) and also have been found to respond differentially to sleep deprivation (SD; Wright et al., 2023), suggesting that there may be sex-specific vulnerabilities to the negative consequences of sleep loss.

Building from these recent studies, we further characterize age-related changes in sleep behavior, test the causal role of sleep disruption as a driver of cognitive decline, and test whether a causal relationship exists between sleep disruption and Tau burden in forebrain synapses in PS19 mice of both sexes. Male and female PS19 mice exhibit early-onset and progressive loss of sleep during the dark phase, a “hyperarousal phenotype,” and further show sleep fragmentation during later stages. Chronic sleep disruption accelerated the decline of hippocampal spatial memory in male but not female PS19 mice. Counter to our expectations, we find no correlative or causal relationship between sleep and forebrain synaptic Tau burden. In contrast, we confirm prior reports that chronic sleep disruption accelerates Tau pathology progression in wake-promoting LC neurons (Zhu et al., 2018). We conclude that sleep disruption is an early symptom of disease progression in this Tau-based disease model and that sleep disruption plays a causal role in cognitive decline in a sex-dependent manner.

## Materials and Methods

### Mice

Animal procedures were all approved by the animal care and use committee of the University of North Carolina Chapel Hill (UNC) and performed according to guidelines set by the US National Institutes of Health. P301S (PS19) male mice were obtained from Dr. Todd Cohen (UNC) and bred with C57BL/6J females purchased from the Jackson Laboratory. C57BL/6J females were allowed to acclimate to the housing for at least 2 weeks before breeding. Experimental animals were bred by crossing wild-type (WT) C57BL/6J females with P301S-positive males and housed in the animal facility until sleep behavioral or molecular analysis. Experiments were performed using 3-, 6-, 9-, and 11-month-old mice. Experiments include WT and P301S littermates of both sexes. WT breeders for our colony were replaced every 3 months with mice supplied by the Jackson Laboratory.

### Sleep phenotyping and behavior analysis

PS19 and C57BL/6J littermates were moved to our wake/sleep behavior satellite facility on a 12 h light/dark cycle (lights on from 7 A.M. to 7 P.M.). Individual mice were housed in 15.5 cm^2^ cages with bedding, food, and water. Before the beginning of data collection, mice were allowed to acclimate to the environment for at least two complete dark cycles. No other animals were housed in the room during these experiments. Sleep and wake behaviors were recorded using a noninvasive home-cage monitoring system, PiezoSleep 2.0 (Signal Solutions), as previously described (Martin et al., 2022). Briefly, the system uses a piezoelectric polymer film to quantitatively assess the sleep/wake cycles, total amount of sleep, and quality of sleep from mechanical signals obtained from breath rate and movement. Specialized software (SleepStats, Signal Solutions) uses an algorithm to discern sleeping respiratory patterns from waking respiratory patterns. Sleep was characterized according to specific parameters in accordance with the typical respiration of a sleeping mouse. Additional parameters were set to identify wake, including the absence of characteristic sleep signals and higher amplitude, irregular signals associated with volitional movements, and even subtle head movements during quiet wake. Data collected from the cage system were binned over specified time periods: 1 h bins to generate a daily sleep trace and 12 h bins for average light- or dark-phase percent sleep or sleep bout lengths. Sleep bout length was determined by an onset occurring when a 30 s interval contained >50% sleep and termination when a 30 s interval had <50% sleep. This algorithm has been validated in adult mice by using EEG, EMG, and visual evaluation (Flores et al., 2007; Donohue et al., 2008; Mang et al., 2014; Yaghouby et al., 2016) and utilized in additional studies (Holth et al., 2019; Lord et al., 2022). REM and NREM sleep amount and bout lengths were estimated based on rapid changes in breath rate coinciding with transitions in vigilance states as reported (Mang et al., 2014; Vanneau et al., 2021). REM and NREM estimates were analyzed using SleepStats version 4.0 (Signal Solutions, Lexington, KY).

### Sleep disruption paradigms

For acute and chronic sleep disruption experiments, animals were brought up from the animal facilities either to our sleep recording satellite facility or behavior satellite room. Animals were acutely sleep-deprived at 6 months via gentle handling for 4 h, as previously described (Suzuki et al., 2013). Briefly, gentle handling consists of light tapping on home cages, rustling or removing bedding, replacing bedding, or movement on a cart. The mice remain in their home cage and are never touched. For chronic sleep disruption, the animal's home cage was placed on top of orbital shakers, which automatically agitated at 110 rpm for 10 s followed by still rest for 99 s (109 s total per cycle), continuing 24 h/d for 30 d. This paradigm has been described by other groups to produce a mild fragmentation of sleep (Zhu et al., 2018; Jones et al., 2019; Lord et al., 2022). Control groups were housed alongside treatment groups atop inactive orbital shakers.

### Morris water maze behavioral paradigm

The water maze was used to assess spatial learning, swimming ability, and vision. The water maze consisted of a large circular pool (diameter, 122 cm) partially filled with water (45 cm deep, 24–26°C), located in a room with numerous visual cues. The procedure involved two phases: a visible platform test and the acquisition of spatial learning in the hidden platform task.

#### Visible platform test

Each mouse was given four trials per day, across 2 d, to swim to an escape platform cued by a patterned cylinder extending above the surface of the water. For each trial, the mouse was placed in the pool at one of four possible locations (randomly ordered) and then given 60 s to find the visible platform. If the mouse found the platform, the trial ended, and the animal was allowed to remain for 10 s on the platform before the next trial began. If the platform was not found, the mouse was placed on the platform for 10 s and then given the next trial. Measures were taken for latency to find the platform and swimming speed via an automated tracking system (Noldus EthoVision).

#### Acquisition of spatial learning in a hidden platform task

Following the visible platform task, mice were tested for their ability to find a submerged, hidden escape platform (diameter, 12 cm). Each mouse was given four trials per day, with 1 min per trial, to swim to the hidden platform. The criterion for learning was an average latency of 15 s or less to locate the platform. Mice were tested until the group reached the criterion, with a maximum of 9 d of testing. When the group reached criterion (on Day 4 in the present study), mice were given a 1 min probe trial in the pool with the platform removed. Selective quadrant search was evaluated by measuring the percent of time spent in the quadrant where the platform (the target) had been located during training, versus the opposite quadrant, and by the number of swim path crosses over the location (diameter, 12 cm) where the platform had been placed during training, versus the corresponding area in the opposite quadrant.

### Postsynaptic density preparation

Male and female mice aged 3, 6, 9, and 11 months were killed; mouse whole cortices or hippocampi were dissected in ice-cold PBS then frozen on dry ice and kept at −80°C until further processing. Frozen mouse cortices were homogenized using 12 strokes from a glass homogenizer in ice-cold homogenization solution [320 mM sucrose, 10 mM HEPES, pH 7.4, 1 mM EDTA, 5 mM Na pyrophosphate, 1 mM Na_3_VO_4_, 200 nM okadaic acid (Roche)]. Brain homogenate was then centrifuged at 1,000 × *g* for 10 min at 4°C to obtain the P1 (nuclear) and S1 (postnuclear) fractions. The S1 fraction was then layered on top of a discontinuous sucrose density gradient (0.8, 1.0, or 1.2 M sucrose in 10 mM HEPES, pH 7.4, 1 mM EDTA, 5 mM Na pyrophosphate, 1 mM Na_3_VO_4_, 200 nM okadaic acid) and then subjected to ultracentrifugation at 82,500 × *g* for 2 h at 4°C. Material accumulated at the interface of 1.0 and 1.2 M sucrose (synaptosomes) was collected. Synaptosomes were diluted using 10 mM HEPES, pH 7.4 (containing protease and phosphatase inhibitors), to restore the sucrose concentration to 320 mM. The diluted synaptosomes were then pelleted by centrifugation at 100,000 × *g* for 30 min at 4°C. Synaptosomes were resuspended in 50 mM HEPES, pH 7.4, and then equal parts of 1× Triton X-100 (both containing inhibitors) were added to each sample. Samples were incubated for 10 min and subjected to ultracentrifugation at 32,000 × *g* for 20 min at 4°C. The supernatant was then removed, resulting in an enriched postsynaptic fraction pellet. The pellet was resuspended in 50 mM HEPES, pH 7.4 (containing inhibitors).

Frozen mouse hippocampi were homogenized 15 times using a 28 ga needle in the ice-cold homogenization solution mentioned above. Brain homogenate was then centrifuged at 1,000 × *g* for 10 min at 4°C to obtain the P1 (nuclear) and S1 (postnuclear) fractions. The S1 fraction was then further centrifuged at 1,000 × *g* for 20 min at 4°C. The resulting S2 (cytosolic) fraction was aspirated, leaving the P2 fraction, which was resuspended in water (containing inhibitors) and incubated (via rotation) for 30–45 min at 4°C. Samples were then centrifuged at 25,000 × *g* for 20 min at 4°C. Crude synaptosomes were then resuspended in 50 mM HEPES pH 7.4, and then equal parts of 1× Triton X-100 (both containing inhibitors) were added. Samples were incubated for 10 min and subjected to ultracentrifugation at 32,000 × *g* for 20 min at 4°C. The supernatant was then removed, resulting in an enriched postsynaptic fraction pellet. The pellet was then resuspended in 50 mM HEPES, pH 7.4 (containing inhibitors).

The protein concentration was determined using the Bradford assay, and Western blot samples were made up based on concentration averages to a known concentration. These protocols allow us to measure soluble Tau localized to PSD fractions.

### Western blot

For Western blot, proteins (5 µg) were separated via sodium dodecyl sulfate–polyacrylamide gel electrophoresis (SDS-PAGE) on a 10% acrylamide gel. After sample separation, the proteins were transferred to 0.2 um nitrocellulose membrane (Bio-Rad) and incubated with 3% BSA (Fisher Bioreagents) blocking buffer (100 mM Tris, pH 7.5, 165 mM NaCl; TBS) for 30 min at room temperature. Membranes were then incubated with primary antibodies (full list provided in Table 1) in a solution of 3% w/v BSA in TBST (100 mM Tris, pH 7.5, 165 mM NaCl, 0.1% v/v Tween 20; TBST) at 4°C overnight. Membranes were then probed with secondary antibodies 1:15,000 IRDye 680RD-conjugated goat anti-mouse (LI-COR Biosciences) and 1:15,000 IRDye 800RD-conjugated donkey anti-rabbit (LI-COR) in 3% w/v BSA in TBST for 1 h at room temperature. The immunoreactivity of all antibody signals was detected simultaneously with a LI-COR Odyssey CLx IR imaging system (LI-COR).

**Table 1.** Western blot antibodies used in PS19 synaptic analysis

Antibody	Species	Company	Concentration
Anti-AT8	Mouse	Thermo Fisher Scientific	1:1,000
Anti-AT180	Mouse	Thermo Fisher Scientific	1:1,000
Anti-AT100	Mouse	Thermo Fisher Scientific	1:1,000
Anti-pS396	Rabbit	Thermo Fisher Scientific	1:1,000
Anti-Tau1	Mouse	EMD Millipore	1:1,000
Anti-K9	Rabbit	Agilent Technologies	1:5,000
Anti-PSD95	Mouse	NeuroMab 75-028	1:1,000,000
Anti-GluA1	Mouse	NeuroMab 75-327	1:1,000
Anti-pS845	Rabbit	EMD Millipore	1:1,000
Anti-pS831	Rabbit	EMD Millipore	1:1,000
Anti-GluA2	Mouse	EMD Millipore	1:1,000
Anti-GluA3	Rabbit	Cell Signaling Technology	1:1,000
Anti-Arc	Mouse	Synaptic Systems	1:1,000
Anti-GluN2A	Rabbit	Cell Signaling	1:1,000
Anti-GluN2B	Rabbit	Cell Signaling Technology	1:1,000
Anti-GluN1	Rabbit	Cell Signaling Technology	1:1,000
Anti-Hsc70	Mouse	EMD Millipore	1:1,000

### Histology

Mice were anesthetized using a nose cap containing isoflurane and pinned in a supine posture, and transcardial perfusion was performed. The system was rinsed with 25 ml of cold PBS and then flushed with 25 ml of 4% paraformaldehyde (PFA) for fixation. Collection of the brain was done via decapitation, and whole brains were stored individually in 4% PFA at 4°C for 48 h and then transferred to PBS at 4°C until slicing and staining. Whole brain samples were coronally sliced (5 μm) to obtain samples of the LC (bregma −5.52 to −5.4). Slices were pretreated, baked, deparaffinized, and hydrated. Thermo, pH 6.0 (#TA-135-HBL), was used for heat-induced epitope retrieval, and then samples were washed with 10% NGS in Tris for 1 h before staining. Slices were then incubated with primary antibodies: 1:250 mouse anti-AT8 (Invitrogen #MN1020), 1:375 rabbit anti-TH (Abcam #ab112, Lot GR3352453-12), and DAPI (RTU). After primary staining, slices were incubated with 1:500 Alexa Fluor 594 goat anti-rabbit IgG (Invitrogen #A11037, Lot 2566328) and 1:500 Alexa Fluor 488 goat anti-mouse IgG (Invitrogen #A11001, Lot 2220848) for visualization. Imaging was done using a Zeiss LSM 780 confocal lens and Zen Black 2.3 software. Fiji imaging software was used to threshold AT8 in all 2 × 2 tile scan images. Percentage AT8 was averaged across each slide for all four slices and both sides of the LC, totaling one AT8 value per animal. Data values were categorized into four groups: wild-type control (WT Ctrl), wild-type chronic sleep disrupted (WT CSD), PS19 control (PS19 Ctrl), and PS19 chronic sleep disrupted (PS19 CSD). One-way ANOVA testing with Bonferroni’s corrections was performed on all group comparisons using GraphPad Prism 10.

### Statistical analysis

All data from sleep behavior experiments were analyzed in Microsoft Excel or GraphPad Prism version 9.1.0 (GraphPad Software). To compare sleep amounts and bouts between PS19 and WT mice, unpaired Student's *t* tests with Bonferroni’s correction for multiple comparisons were used. Behavioral tests were performed by experimenters blinded to mouse genotype. StatView (SAS Communities) was used for data analysis. Repeated-measures ANOVA was used to determine the effects of genotype, treatment, and sex. Separate analyses were then conducted to determine the effects of genotype within each treatment group and sex. Post hoc comparisons were conducted using Fisher's protected least significant difference (PLSD) tests only when a significant *F* value was found in the ANOVA. Within-genotype comparisons were conducted to determine quadrant selectivity in the water maze. All data are presented as mean ± SEM, *p < 0.05, **p < 0.01, ***p < 0.001.

## Results

### Hyperarousal phenotypes emerge as an early symptom of disease progression in PS19 mice, particularly in females

**Figure 1.**
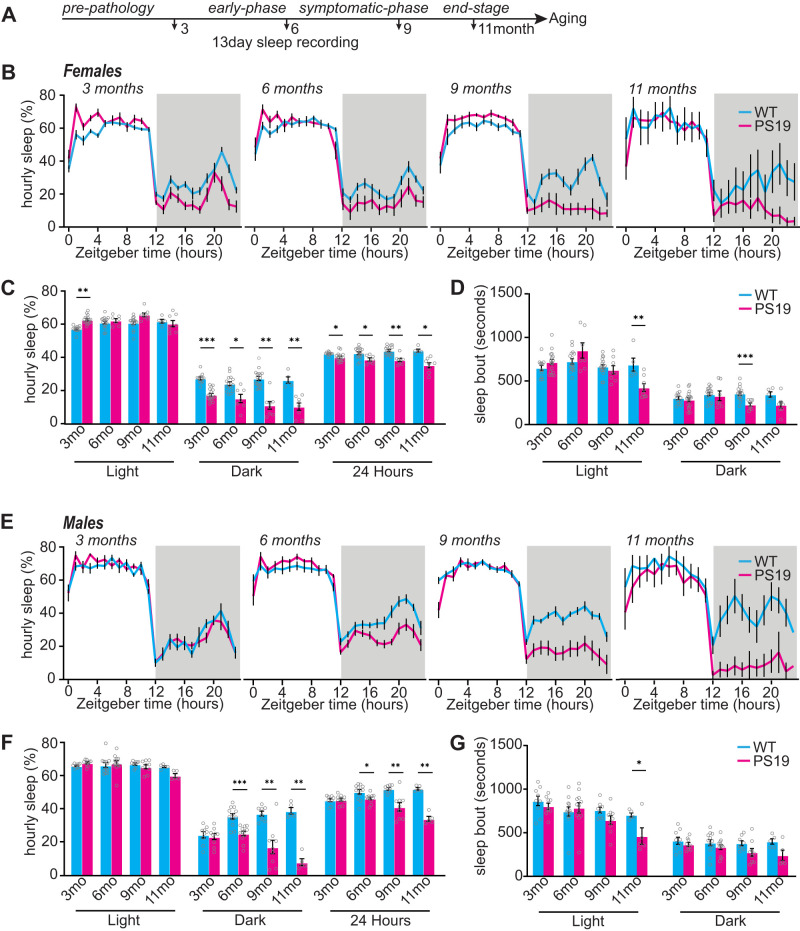
PS19 mice have decreased sleep that worsens with age. ***A***, Experimental design. ***B***, Twenty-four hours of tracing the average hourly sleep of female WT (blue line) PS19 (pink line) mice at 3 months (prepathology), 6 months (early-phase), 9 months (symptomatic-phase), and 11 months (end-stage). The gray bars in sleep traces indicate a dark phase. ***C***, ***D***, Quantification of average hourly sleep (***C***) and sleep bout length in seconds (***D***). Data separated into 12 h of dark and light phases. ***E***, Twenty-four hours of tracing the average hourly sleep of male WT (blue line) and PS19 (pink line) mice at 3, 6, 9, and 11 months. ***F***, ***G***, Quantification of average hourly sleep (***F***) and sleep bout length in seconds (***G***). Data separated into 12 h of dark and light phases. *N* = 5–17/age/sex/genotype. *p < 0.05, **p < 0.01, ***p < 0.001. Unpaired two-tailed Student's *t* test. Error bars indicate ± SEM. Estimated NREM and REM sleep % time in state and bout lengths are presented in Extended Data Figure 1-1 (females) and Extended Data Figure 1-2 (males).

Sleep disturbances are widespread in AD patients and have been reported in various animal models of AD, including PS19 Tau transgenic AD/FTD mouse model (Holth et al., 2017; C. Wang and Holtzman, 2019; Kam et al., 2023; Morrone et al., 2023). In order to better understand the causal links between Tau hyperphosphorylation and sleep disruption and to ask whether there may be sex-specific vulnerabilities to sleep disruption, we first investigated the onset and progression of sleep disruption in PS19 mice by measuring sleep behavior at 3 months (prepathology), 6 months (early pathology), 9 months (late pathology, early atrophy), and 11 months (late pathology, late atrophy; Yoshiyama et al., 2007; Dejanovic et al., 2018). Sleep behavior was examined in male and female PS19 mice, in comparison with WT littermates, using the PiezoSleep Mouse Behavioral Tracking System (Signal Solutions). This system uses piezoelectric sensors to measure home-cage mouse motion and breathing to provide accurate measurements of total sleep time and bout lengths, achieving ∼95% accuracy when compared with EEG/EMG, and has further been validated with video recordings (Flores et al., 2007; Donohue et al., 2008; Mang et al., 2014; Yaghouby et al., 2016). Mice were maintained on a 12 h light/dark cycle and allowed a 2 d acclimation period to the home-cage recording system prior to data collection. Average daily total sleep amount and bout lengths were calculated from 13 consecutive days, providing robust measures of typical daily sleep patterns for each individual (Fig. 1*A*). As expected, all mice examined spent more time sleeping during the light phase and were more active during the dark phase. Overall, male and female WT and PS19 mice show comparable total sleep amount in the light phase at 3, 6, 9, and 11 months, with the exception of PS19 females that showed a small but significant increase in light-phase sleep amount at 3 months (Fig. 1*B*,*C*,*E*,*F*). In contrast, sleep amount in the dark/active phase was significantly reduced in PS19 females as early as 3 months and progressed with age through 11 months (Fig. 1*B*,*C*). Even with the small increase in light-phase sleep in females at 3 months, the loss of dark-phase sleep resulted in a significant decrease in total daily (24 h) sleep compared with WT controls at all ages (Fig. 1*C*). Males showed a similar reduction in dark-phase sleep that only became apparent at 6 months of age and was progressive through 11 months, again resulting in a significant reduction in total 24 h of sleep (Fig. 1*F*). Summarized sleep measures for mice in Figure 1 are shown in Extended Data Table 1-1. We refer to this selective loss of sleep during the dark phase as a “hyperarousal” phenotype. Sleep bout lengths, a crude measure of sleep quality, showed some significant reductions or compelling trends in males and females at 9–11 months, signifying reduced sleep quality with age and disease progression (Fig. 1*D*,*G*), consistent with previous reports (Holth et al., 2017; Kam et al., 2023). Together, we find that dark-phase hyperarousal and consequent loss of total daily sleep is an early symptom in PS19 mice and females particularly and that sleep quality continues to decline with age.

10.1523/ENEURO.0004-24.2024.t1-1 Table 1-1 Summary sleep measures from Figure 1. Sleep amount: % of time spent sleeping in the light or dark phase. Average sleep bout length (s) in light and dark phase. Download Table 1-1, XLSX file.

10.1523/ENEURO.0004-24.2024.f1-1 Figure 1-1 PS19 female mice exhibit progressive decrease in REM and NREM sleep. (A) 24hr trace of NREM sleep in female WT (blue line) PS19 (pink line) mice at 3, 6, 9, 11 months. Grey bars in sleep traces indicate dark phase. (B and C) Quantification of average hourly NREM sleep amount (B) and NREM sleep bout length in seconds (C). (D) 24hr trace of REM sleep in female WT (blue line) PS19 (pink line) mice at 3, 6, 9, 11 months. Grey bars in sleep traces indicate dark phase. (E and F) Quantification of average hourly REM sleep amount (E) and REM sleep bout length in seconds (E). Data separated into 12hrs of dark and light phases. N = 5-17/age/genotype. *p < 0.05, **p < 0.01, ***p < 0.001 Unpaired two-tailed student’s t-test. Error bars indicate ± SEM. Download Figure 1-1, TIF file.

**Figure 2.**
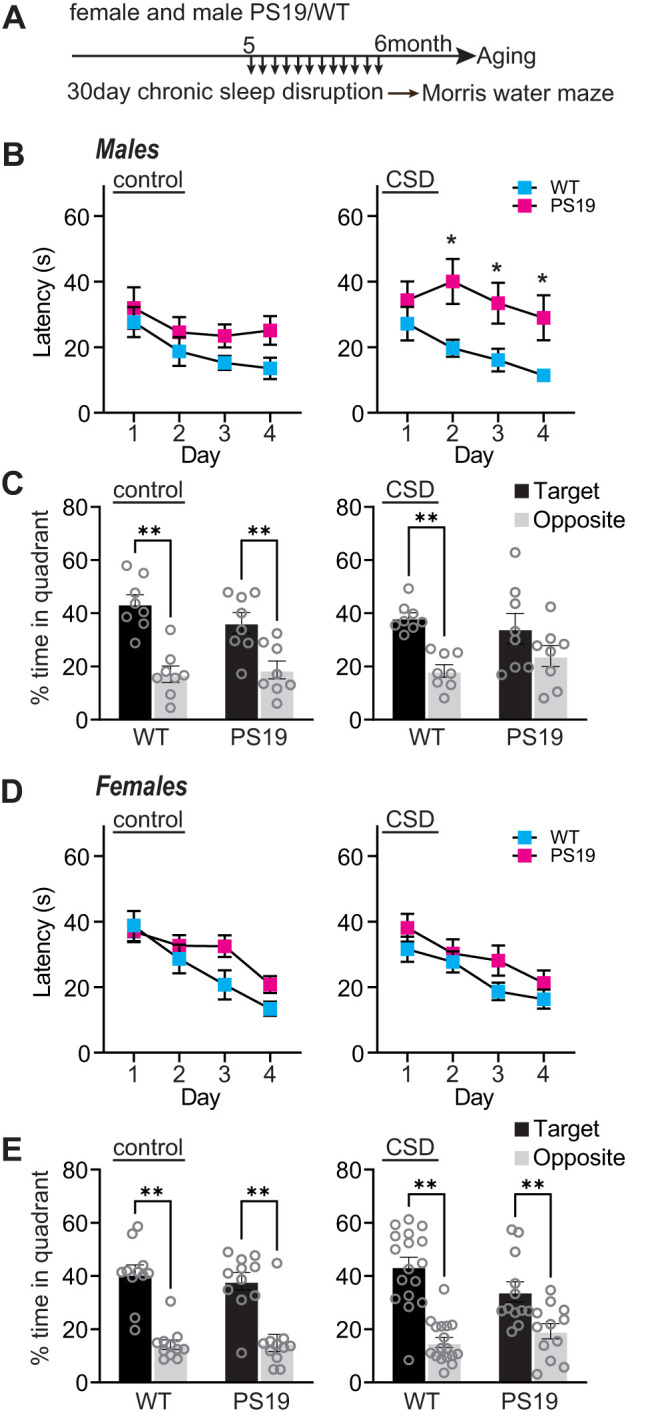
Chronic sleep disruption accelerates cognitive decline in males. ***A***, Experimental design. ***B***, Males, Acquisition of spatial learning. Escape latencies during training in control and CSD treated mice. ***C***, Males, Spatial memory retention during 1 min of probe trial, time in target or opposite quadrant in control and CSD treated mice. Target indicates the quadrant where the platform had been located, versus the opposite quadrant. ***D***, Females, Acquisition of spatial learning. Escape latencies during training in control and CSD treated mice. ***E***, Females, Spatial memory retention during 1 min of probe trial, time in target or opposite quadrant in control and CSD treated mice. *N* = 8–17 per group (sex, genotype, treatment). ***B***, ***D***, Data are means(±SEM) of four trials per day. ***C***, ***E***, Data are means (+SEM). *p < 0.05, **p < 0.01.

10.1523/ENEURO.0004-24.2024.f1-2 Figure 1-2 PS19 male mice exhibit progressive decrease in REM and NREM sleep. (A) 24hr trace of NREM sleep in male WT (blue line) PS19 (pink line) mice at 3, 6, 9, 11 months. Grey bars in sleep traces indicate dark phase. (B and C) Quantification of average hourly NREM sleep amount (B) and NREM sleep bout length in seconds (C). (D) 24hr trace of REM sleep in male WT (blue line) PS19 (pink line) mice at 3, 6, 9, 11 months. Grey bars in sleep traces indicate dark phase. (E and F) Quantification of average hourly REM sleep amount (E) and REM sleep bout length in seconds (E). Data separated into 12hrs of dark and light phases. N = 5-17/age/genotype. *p < 0.05, **p < 0.01, ***p < 0.001 Unpaired two-tailed student’s t-test. Error bars indicate ± SEM. Download Figure 1-2, TIF file.

### Estimation of progressive REM and NREM sleep phenotypes in PS19 mice of both sexes

Piezoelectric-based scoring of mouse wake and sleep states is based on the detection of characteristic mechanical signals related to mouse breathing, only apparent in the absence of motions associated with wake, even quiet wake (called two-stage scoring). Recent advances have shown that NREM and REM states can be estimated noninvasively through rapid changes in breath rate that accompany vigilance state transitions (Mang et al., 2014; Vanneau et al., 2021). To extend our sleep phenotyping of male and female PS19 mice (Fig. 1), we additionally used PiezoSleep to estimate progressive changes in wake, REM, and NREM in comparison with WT littermates (called three-stage scoring). PS19 mice of both sexes display decreases in REM and NREM, particularly in the later stages of progression, consistent with prior reports of PS19 mice using EEG (Holth et al., 2017; Kam et al., 2023; Extended Data Figs. 1-1, 1-2). Females displayed subtle changes in daily NREM amount and distribution at 3 months, whereas REM sleep was reduced starting at 6 months. Interestingly, males exhibited no change in NREM measure at 3 months, while showing an increase in estimated REM sleep during the light phase at 3 and 6 months before REM sleep significantly declined at 11 months (Extended Data Figs. 1-1, 1-2). These findings suggest that sex-specific changes in sleep amount and physiology are an early-stage, progressive symptom in PS19 mice, consistent with early-stage changes in sleep microarchitecture recently reported (Kam et al., 2023). The summarized estimated NREM/REM sleep measures are shown in Extended Data Table 1-2.

10.1523/ENEURO.0004-24.2024.t1-2 Table 1-2 Summary of estimated NREM and REM sleep measures from extended data Figures 1-1 and 1-2. Estimated NREM/REM Sleep amount: % of time spent sleeping in the light or dark phase. Average estimated NREM/REM sleep bout length (s) in light and dark phase. Download Table 1-2, XLSX file.

### Chronic sleep disruption accelerates the decline of hippocampal-dependent spatial learning and memory in male PS19 mice

Early-onset and sex-specific changes in sleep amount and composition at 3–6 months of age, (Fig. 1; Extended Data Figs. 1-1, 1-2; Kam et al., 2023) suggest that sleep disruption may play a causal, and sex-specific, role in age-related cognitive decline. Indeed, PS19 mice are known to display age-related deficits in learning and memory, which can be accelerated via chronic sleep disruption treatments (Zhu et al., 2018). However, whether sleep disruption drives sex-specific effects on cognitive performance has not been investigated. Here, we investigated sex-specific cognitive effects of a 30 d CSD, via Morris water maze (MWM; Fig. 2*A*). CSD was achieved by placing the home cage on top of an orbital shaker set to agitate at 110 rpm for 10 s every 109 s (99 s rest) for 24 h/d, providing a mild but regular stimulus demonstrated to cause sleep fragmentation (Zhu et al., 2018; Jones et al., 2019; Lord et al., 2022); the control groups were housed in the same room and placed onto identical orbital shakers that were left off. As male and female PS19 mice exhibit sleep disruption by 6 months (Fig. 1), an age where early-onset pathology and behavioral symptoms also begin to be apparent (Yoshiyama et al., 2007; Dejanovic et al., 2018), we were motivated to investigate the cognitive consequences of CSD leading up to this potentially vulnerable age point. Therefore, 5-month-old male and female WT and PS19 mice underwent 30 d CSD treatment followed by cognitive assessment via MWM at age ∼7 months (Fig. 2*A*). Note that this age is well in advance of cognitive impairment previously demonstrated in PS19 using MWM at 9 months of age (Dejanovic et al., 2018).

In the first phase of this testing, mice were placed into the water tank with the escape platform visible. Because PS19 mice are known to undergo age-related motor impairments (Patel et al., 2022), we first confirmed that all mice tested were able to swim and locate the visible platform. All our mice were able to perform the task (data not shown); no mice were excluded from further testing. In the next phase of the experiment, the platform was hidden, and mice underwent 4 d of training, four trials per day, to learn the location of the hidden platform based on spatial cues placed around the water maze. All our mice showed the expected reduction in escape latency (finding the hidden platform) with repeated days of training, with the exception of PS19 males exposed to CSD that showed significantly longer escape latencies than WT siblings, and no clear trend to reduce latency with multiple days of training, indicating that male CSD-PS19 mice show impairments in learning in this spatial task (Fig. 2*B*,*D*). In the final phase of the test, the hidden platform was removed, the mice were returned to the water maze, and their time spent searching in the former location of the platform was measured, a readout of spatial memory retention. Consistent with the above results, all our mice spent significantly more time in the target quadrant of the maze than in the opposite quadrant, indicating intact spatial memory, with the exception of male CSD-PS19 mice, indicating impaired memory retention in this group (Fig. 2*C*,*E*). Previous studies have shown that PS19 mice eventually develop impairments in the Morris water maze at 9 months (Dejanovic et al., 2018); however, at the age tested here (6–7 months), PS19 mice of both sexes in the control group performed comparably to WT littermates. Therefore, these results show that CSD treatment was able to accelerate the onset of cognitive decline in male PS19 mice, whereas female PS19 and WT littermates were found to be resilient to these negative effects. This result motivated us to further examine the mechanisms linking sleep loss with disease progression and the basis of sex-specific vulnerability or comparative resilience, centered on the putatively vulnerable 6 months of age.

### Tau burden in the forebrain synapse fraction begins to appear at 6 months in PS19 mice

**Figure 3.**
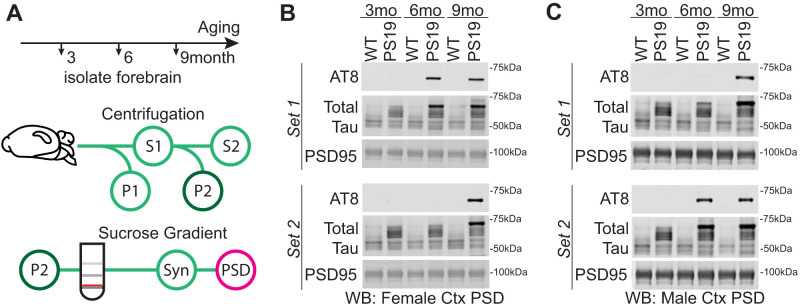
Tau in the cortex postsynaptic density (PSD). ***A***, Subcellular fractionation technique to isolate PSD. The red line indicates the synaptosome fraction used to further isolate the PSD. ***B***, ***C***, Western blot analysis of cortex PSD samples showing soluble phosphorylated Tau (AT8) accumulation with age in PS19 and WT females (***B***) and males (***C***). Western blots also include total Tau and PSD95, a protein enriched in the PSD.

Forebrain synapses have been shown to be modified during sleep, mediating the benefits of sleep on cognitive function (Tononi and Cirelli, 2014; Diering et al., 2017). Tau is normally an axonal protein but has been shown to be present in dendrites and postsynaptic density (PSD) (Ittner and Ittner, 2018), where Tau contributes to synapse dysfunction and cognitive decline. Note that this synaptic targeted Tau is likely representing a distinct pool of soluble Tau species, separate from the insoluble Tau found in large neurofibrillary tangles (Yoshiyama et al., 2007). The PSD is a fraction highly enriched with synaptic proteins and is amenable to biochemical isolation (Diering et al., 2017). A recent publication has shown that soluble phospho-Tau (AT8 epitope) becomes enriched in the hippocampal PSD fraction of 9-month-old PS19 females (Dejanovic et al., 2018). Based on this finding, we speculated that AT8-positive Tau would also be detected in PSD fractions isolated from the whole cortex in an age-dependent manner. We used biochemical fractionation and Western blot to assess the presence of soluble, hyperphosphorylated Tau (AT8 positive) in the PSD fractions isolated from the whole cortex of WT and PS19 mice at 3, 6, and 9 months (Fig. 3*A*). No AT8-Tau was detected in WT littermates at any age (Fig. 3*B*). Consistent with the prior report (Dejanovic et al., 2018), AT8-Tau was detected in cortex PSD fractions in every PS19 mouse examined at 9 months, whereas no PS19 mice were AT8-positive at 3 months; example Western blots are shown in Figure 3, *B* and *C*. Interestingly, variable synaptic Tau burden was seen in males and females at 6 months in the cortex (Fig. 3*B*,*C*; Fig. 3, compare “Set 1” and “Set 2”). Approximately 50% of PS19 mice show AT8-Tau in cortex PSD at 6 months, and the remainder, we conclude, have not yet developed synaptic Tau burden. This observation further suggested that 6 months may be an age of particular vulnerability, where forebrain synaptic Tau burden is first beginning to appear and where sleep disruption is already significant in both sexes (Fig. 1).

### Sleep disruption drives Tau burden in locus ceruleus but is not causal in driving Tau burden in forebrain synapses

**Figure 4.**
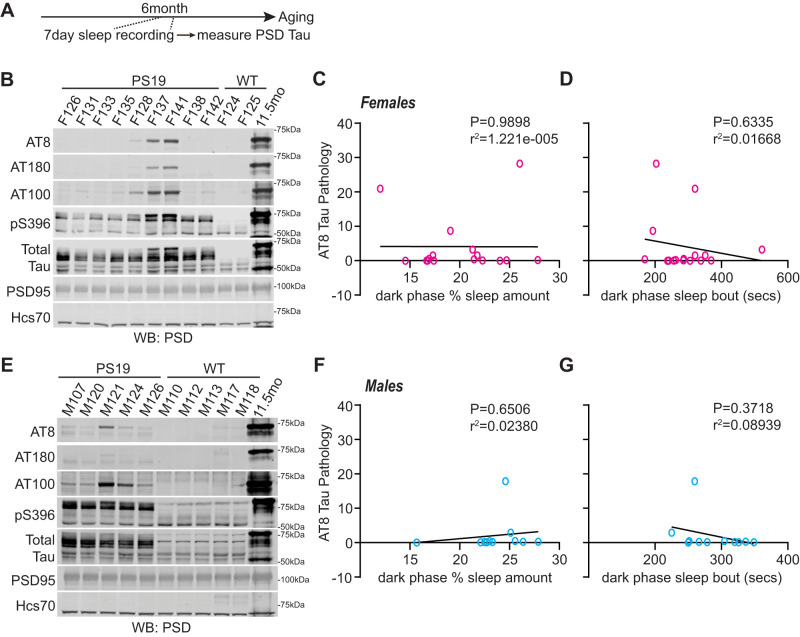
Decreased sleep amount in PS19 Tau tg mice is not predictive of AT8-Tau pathology in the cortex. ***A***, Experimental design. ***B***, Western blot analysis of AT8, AT180, AT100, pS396, total Tau, and PSD95 in 6-month-old (early-phase) PS19 females. ***C***, ***D***, Correlation of AT8-Tau hyperphosphorylation expression in the cortex of PS19 females with average dark-phase hourly sleep (***C***) or sleep bout length in seconds (***D***). Sleep data were separated into 12 h of dark and light phases. Dark-phase sleep is represented here. *N* = 16 PS19 females. ***E***, Western blot analysis of AT8, AT180, AT100, pS396, total Tau, and PSD95 in 6-month-old PS19 males. ***F***, ***G***, Correlation of AT8-Tau hyperphosphorylation expression in the cortex of PS19 males with average dark-phase hourly sleep (***F***) or sleep bout length in seconds (***G***). Sleep data were separated into 12 h of dark and light phases. Dark-phase sleep is represented here. *N* = 11. All antibodies normalized to loading control. No significance (Pearson’s correlation). See Extended Data Figure 4-1 for further correlation analysis between sleep metrics and phosphorylated Tau.

Because dark-phase sleep disruption (hyperarousal) is clearly apparent at 6 months of age in both males and females, well in advance of the robust and widespread Tau pathology at 9 months (Dejanovic et al., 2018; Fig. 3), we hypothesized that sleep disruption may be a direct driver of Tau hyperphosphorylation in the forebrain synapse, as has been demonstrated in the brainstem LC neurons (Zhu et al., 2018). As a first step to test this hypothesis, we examined whether variations in sleep behavior in PS19 mice at 6 months are predictive of phospho-Tau in forebrain PSD fractions. Cohorts of 6-month-old male and female PS19 mice underwent a 7 d of sleep recording, followed by sacrifice, isolation of the whole cortex PSD fraction, and Western blot analysis of several markers of Tau pathology. In order to provide a reliable standard for which to quantitatively compare Tau burden in our 6-month-old individuals, we pooled whole cortex PSD factions from 4× 11.5-month-old (late-stage disease) PS19 mice of both sexes and included this late-stage pooled PSD material in our Western blots. Synaptic Tau burden in 6-month-old mice, assessed through Western blot of a number of Tau epitopes including AT8, was then normalized to this late-stage 11-month standard, and synaptic Tau burden was correlated with dark-phase sleep measures for each mouse. Counter to our hypothesis, we found no statistical correlation (Pearson's) between dark-phase sleep amount or bout length and synaptic AT8-Tau hyperphosphorylation in the cortex in females (Fig. 4*A*–*C*) or males (Fig. 4*D*–*F*). No other Tau epitopes were found to correlate with dark-phase sleep measures (Extended Data Fig. 4-1). These results show that sleep behavior immediately before sacrifice in 6-month-old PS19 mice is not predictive of synaptic Tau burden in the cortex.

10.1523/ENEURO.0004-24.2024.f4-1 Figure 4-1 Decreased sleep amount in PS19 Tau tg mice is not predicative of AT8 Tau pathology in the cortex (continued). Western blots analysis of AT180, AT100, pS396 (see Figure 3) correlated to dark phase sleep measures in 6-month (early phase) PS19 females and males. (A and B) Correlation analysis of AT180, AT100, pS396 Tau pathology expression in the cortex of PS19 females with average dark phase hourly sleep (A) or sleep bout length in seconds (B). N = 16 PS19 females. (C and D) Correlation analysis of AT180, AT100, pS396 Tau pathology expression in the cortex of PS19 males with average dark phase hourly sleep (C) or sleep bout length in seconds (D). N = 11 PS19 males. All antibodies normalized to loading control. No significance (Pearson correlation). Download Figure 4-1, TIF file.

**Figure 5.**
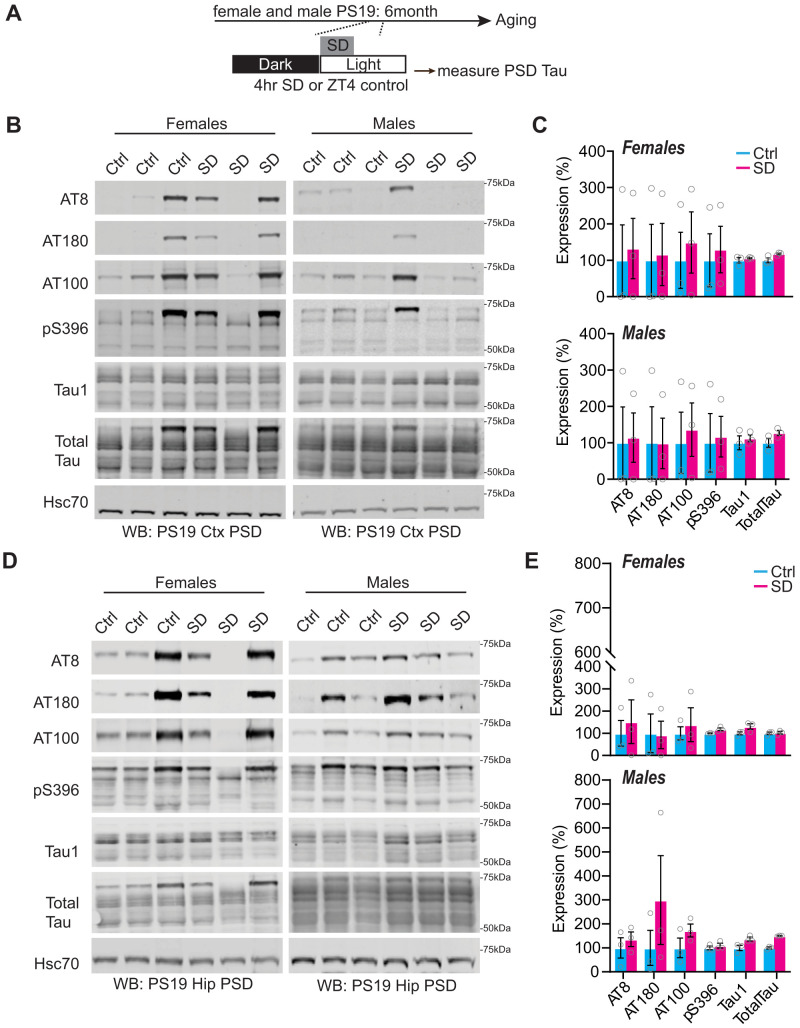
Synaptic Tau is not increased in PS19 mice after acute sleep deprivation in the cortex and hippocampus. ***A***, Experimental design. ***B***, Western blot analysis of AT8, AT180, AT100, pS396, Tau1, and total Tau cortical expression in 6-month-old (early pathology) PS19 females and males. ***C***, Quantification of cortical synaptic Tau proteins in PS19 females and males. *N* = 3 control; 3 CSD per sex. ***D***, Western blot analysis of AT8, AT180, AT100, pS396, Tau1, and total Tau hippocampal expression in 6-month-old (early pathology) PS19 females and males. ***E***, Western blot analysis of AT8, AT180, AT100, pS396, Tau1, and total Tau hippocampal expression in 6-month-old (early pathology) PS19 females and males. *N* = 3 control; 3 SD per sex. All antibodies normalized to loading control and then normalized to the control group. Unpaired two-tailed Student's *t* test between the control and treatment groups. Error bars indicate mean ± SEM.

**Figure 6.**
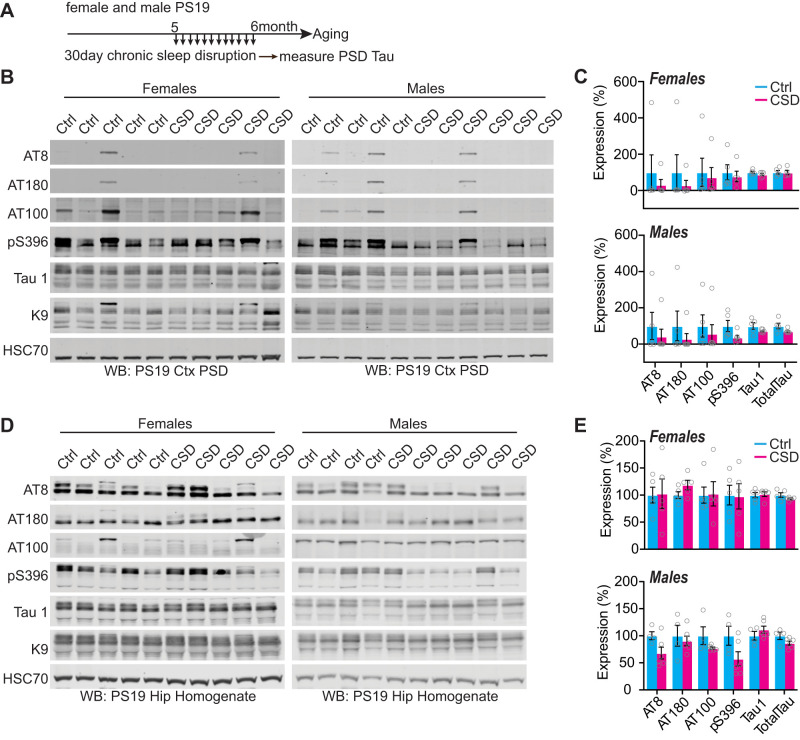
Synaptic Tau is not increased in PS19 mice after chronic sleep disruption in the cortex and hippocampus. ***A***, Experimental design. ***B***, Western blot analysis of AT8, AT180, AT100, pS396, Tau1, and total Tau in cortical PSD fractions of 6-month-old (early pathology) PS19 females and males. ***C***, Quantification of cortical synaptic Tau proteins in PS19 females and males. *N* = 5 control; 5 CSD females; *N* = 5 control; 6 CSD males. ***D***, Western blot analysis of AT8, AT180, AT100, pS396, Tau1, and total Tau in the hippocampi of 6-month-old PS19 females and males. ***E***, Quantification of hippocampal Tau proteins in PS19 females and males. *N* = 5 control; 5 CSD females; *N* = 4 control; 6 CSD males. All antibodies normalized to loading control and then normalized to the control group. Unpaired two-tailed Student's *t* test between the control and treatment groups. Error bars indicate mean ± SEM.

**Figure 7.**
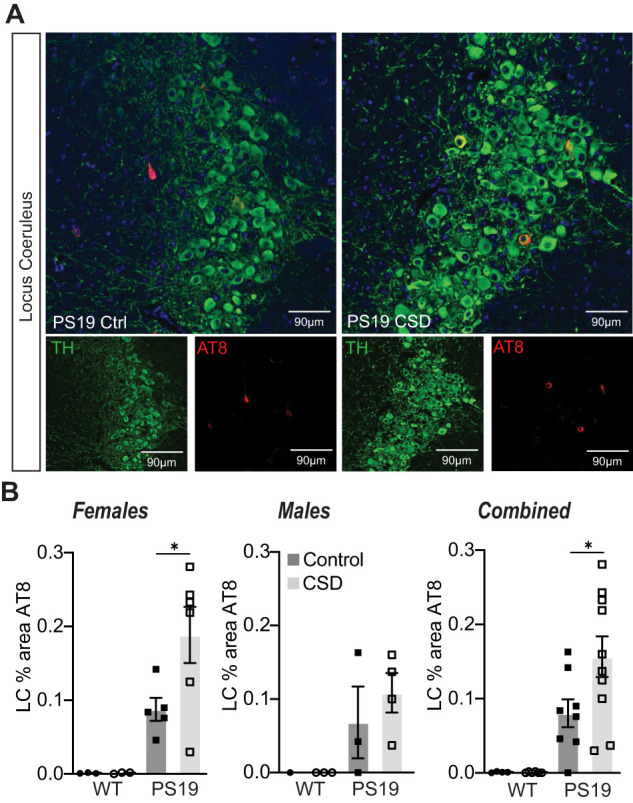
Chronic sleep disruption is a driver of Tau pathology in LC. ***A***, Histology of locus ceruleus sections from PS19 mice under control or following 30 d of CSD treatment. Slices stained with TH to mark the noradrenergic neurons and AT8 to mark the Tau pathology. Example images from female PS19 mice. ***B***, % area positive for AT8 in females, males, or combined. No AT8 signal was detected in WT littermates; therefore, statistical analysis was only conducted comparing control and CSD treatment in PS19 animals. CSD drove a significant increase in AT8 % area compared with control treatment in PS19 females or combined sexes. PS19 males showed a trend to increase. *p < 0.05 unpaired two-tailed Student's *t* test between the control and treatment groups for PS19 genotype. Error bars indicate mean ± SEM.

To further examine the relationship between sleep disruption and forebrain synaptic Tau burden, we tested whether experimentally induced acute 4 h of total sleep deprivation (SD4) or 30 d of CSD was causal in driving Tau hyperphosphorylation at the synapse at the vulnerable 6 months of age. Acute SD4 addresses the immediate response to sleep loss, while CSD investigates the accumulated consequences of sustained sleep loss/fragmentation. We, therefore, performed SD4 on male and female PS19 mice at 6 months of age and CSD experiments from 5 to 6 months (as in Fig. 2) and measured synaptic Tau burden immediately following treatment in comparison with undisturbed controls. SD4 was achieved through gentle handling to keep mice awake for 4 h starting from light onset (zeitgeber time, ZT0-4); the control groups were left undisturbed and killed at the same time of the day (ZT4) (Fig. 5*A*). CSD was achieved by placing the home cage on top of on an orbital shaker for 30 d, as described above; the control groups were housed in the same room and placed onto identical orbital shakers that were left off (Fig. 6*A*). Control or CSD treated mice were all killed at ZT4. Compared to undisturbed controls, SD4 treatment had no effect on synapse Tau hyperphosphorylation in the cortex or hippocampus in PS19 mice of either sex (Fig. 5*B*–*E*). effect on cortex or hippocampus synaptic Tau burden Surprisingly, the more sustained CSD treatment also had no measurable in comparison with control treatments in either sex (Fig. 6*B*–*E*). In contrast to these findings from the forebrain, we were able to replicate a previous finding that CSD treatment significantly increases Tau burden in wake-promoting noradrenergic neurons of the LC (Zhu et al., 2018). Following CSD treatment, WT and PS19 littermates of both sexes were killed and prepared for histology and costained LC marker TH with phospho-Tau AT8. As expected, no AT8 staining was observed in WT samples. Consistent with the prior report, CSD treatment significantly increased AT8 staining in LC compared with control. When we separated the sexes, we observed this effect was significant in females, while males showed a comparable and compelling trend that was not statistically significant (Fig. 7*A*,*B*). Based on these findings, we conclude that sleep disruption is a direct driver of Tau pathology in wake-promoting LC neurons, as reported previously (Zhu et al., 2018), but is not a direct driver of synaptic Tau burden in the forebrain.

### Chronic sleep disruption in PS19 drives female-specific adaptation in the hippocampus

**Figure 8.**
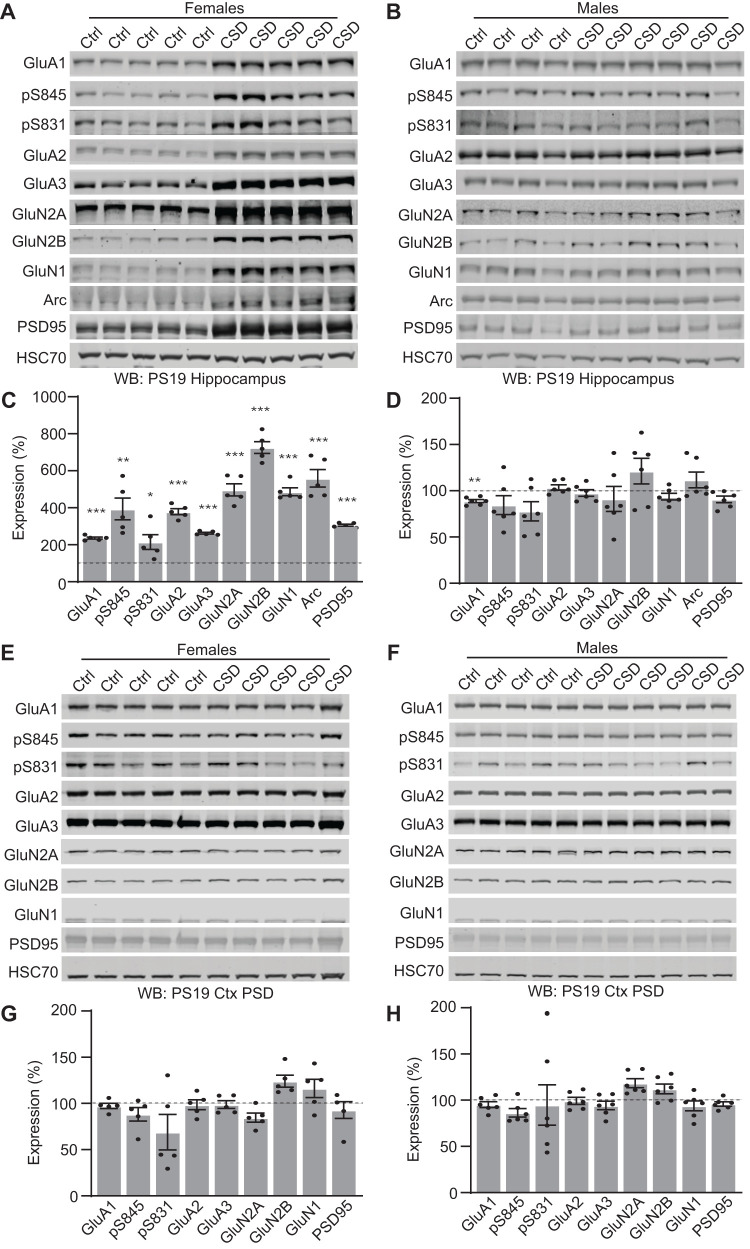
Chronic sleep disruption leads to changes in hippocampal synaptic protein expression in PS19 females but not males. ***A***, ***B***, Western blot analysis of hippocampal synaptic receptor expression in 6-month-old PS19 females (***A***) and males (***B***). ***C***, Quantification of hippocampal protein expression in PS19 females. *N* = 5 control; 5 CSD. ***D***, Quantification of hippocampal protein expression in PS19 males. *N* = 4 control; 6 CSD. All antibodies normalized to loading control and then normalized to the control group. Unpaired two-tailed Student's *t* test between the control and treatment groups for each genotype. *p < 0.05, **p < 0.01, ***p < 0.001. Error bars indicate mean ± SEM. ***E***, ***F***, Western blot analysis of cortical synaptic protein expression in 6-month-old PS19 females (***E***) and males (***F***). ***G***, Quantification of cortical synaptic protein expression in PS19 females. *N* = 5 control; 5 CSD. ***H***, Quantification of cortical synaptic protein expression in PS19 males. *N* = 4 control; 6 CSD. All antibodies normalized to loading control and then normalized to the control group. Unpaired two-tailed Student's *t* test between the control and treatment group for each genotype. Error bars indicate mean ± SEM.

Chronic sleep disruption has been shown to negatively impact AD pathogenesis and may leave synapses vulnerable to disease over time (C. Wang and Holtzman, 2019; Morrone et al., 2023). While sleep disruption did not directly drive forebrain synaptic Tau hyperphosphorylation, we reasoned that the presence of synaptic Tau in PS19 mice may alter how synapses adapt to sleep loss and thereby synergize with sleep loss to drive cognitive impairments. Therefore, we investigated whether cortex and hippocampus synaptic protein expression was altered in the male and female PS19 mice exposed to CSD treatment for 30 d from 5 to 6 months of age (as in Fig. 2*A*) in comparison with undisturbed controls. We focused on AMPA- and NMDA-type glutamate receptors as these receptors are major mediators of synaptic strength and plasticity (Diering and Huganir, 2018). In addition, we examined the phosphorylation of AMPAR subunit GluA1 at two sites, S831 and S845, known to be involved in synaptic plasticity (Diering et al., 2016; Diering and Huganir, 2018). Interestingly, in response to CSD treatment, female PS19 mice showed a striking increase in the expression of hippocampal AMPA and NMDA receptor subunits, GluA1 phosphorylation, and immediate early gene Arc and synaptic scaffold protein PSD95 in comparison with undisturbed controls (Fig. 8*A*,*C*). This response was completely absent in male PS19 mice (Fig. 8*B*,*D*). In contrast to our findings from the hippocampus, no statistical differences were observed in the cortex of male or female PS19 mice exposed to CSD (Fig. 8*E*–*H*). These results suggest that PS19 females, and not males, respond to chronic sleep disruption with a compensatory increase in synaptic protein expression in the hippocampus but not the cortex.

## Discussion

Previous studies have shown that sleep disruption is present in human AD patients and animal models and that sleep disruption can contribute to Tau accumulation and spread (Zhu et al., 2018; C. Wang and Holtzman, 2019; Holth et al., 2019; Morrone et al., 2023). It is unknown whether the presence of synaptic Tau may affect restorative synapse remodeling during sleep or whether sleep disruption drives Tau pathology in the synapse. Synapses play a significant role in the restorative benefits of sleep (Tononi and Cirelli, 2014; de Vivo et al., 2017; Diering et al., 2017; Bruning et al., 2019; Noya et al., 2019). In the current study, we examined the age-related progression of sleep phenotypes in relation to forebrain synaptic Tau burden in both male and female PS19 mice. We show that PS19 mice exhibit an early-onset hyperarousal phenotype: reduced sleep time in the dark phase in comparison with WT littermates. Hyperarousal showed earlier onset in females, and in both sexes, was progressive with age. Based on these data, we hypothesized that CSD would drive acceleration of cognitive decline in hippocampal-dependent learning and memory in a sex-specific manner. Indeed, the results of the Morris water maze show that chronic sleep disruption resulted in a clear acceleration of cognitive decline in male PS19 mice, whereas females were found to be resilient. At 3 months, there is no AT8-positive Tau detected in the synapse fraction in any mice tested, whereas at 9 months, synaptic AT8 is detected in all PS19 mice tested. At 6 months, approximately 50% of PS19 mice show synaptic AT8, indicating that this age may be a vulnerable time point where synapse-associated Tau hyperphosphorylation in the forebrain is emerging. We hypothesized that sleep disruption may be a direct driver of synaptic Tau hyperphosphorylation at this vulnerable 6-month time point. However, counter to our hypothesis, we were not able to establish any correlation or causal relation between sleep and forebrain synaptic Tau burden, unlike in the LC (Zhu et al., 2018; Fig. 7). Strikingly, after CSD, PS19 females showed increased hippocampal synaptic protein expression not seen in males. Our findings highlight two questions of interest for future studies. First, what mechanisms drive synaptic Tau hyperphosphorylation, and does the promotion of sleep mitigate the consequences of synaptic Tau? Second, what mechanisms differentiate male and female responses to sleep disruption in AD, and what are the therapeutic implications of sex differences in the treatment of AD?

### Tau, sleep deficits, and hyperarousal

Does Tau pathology drive sleep disruption, or does sleep disruption drive Tau pathology? A previous study used EEG to demonstrate that male PS19 mice show a progressive decline in sleep amount and bout length (sleep fragmentation) at 9–11 months (Holth et al., 2017). A more recent study further demonstrates a significant, progressive decline in sleep in PS19 of both sexes and changes in sleep microarchitecture (Kam et al., 2023). In the former study, sleep amount was found to negatively correlate with AT8-Tau pathology in sleep-promoting regions of the brainstem, the sublaterodorsal area, and the parafacial zone, but not in the cortex, suggesting that late-stage sleep fragmentation may be caused by Tau pathology in these regions (Holth et al., 2017). Our current data using noninvasive piezoelectric-based measures of home-cage sleep indicate that PS19 mice of both sexes exhibit sleep fragmentation at 9–11 months (Fig. 1), consistent with the prior study (Holth et al., 2017). In addition, we see alterations in sleep behavior occurring earlier than previously reported in the form of a selective dark-phase hyperarousal that was significant in females as young as 3 months and became apparent in males by 6 months. This hyperarousal phenotype became progressively worse, which is supported by a recent study showing an increase in the arousal index of aged (>10 months) male and female PS19 mice (Kam et al., 2023). Moreover, males showed unique early and progressive changes in estimated REM sleep (Extended data Fig. 1-2), indicating that changes in sleep amount and architecture are an early indication of disease progression. Early-stage hyperarousal may not have been noted in the prior EEG-based study because the dark phase was not analyzed separately and females, with their earlier onset, were not included (Holth et al., 2017). Although hyperarousal was limited to the dark/active phase, it still resulted in a significant decrease in total daily sleep at 3 months in females and 6 months in males and was progressive thereafter. Because hyperarousal is apparent well in advance of robust Tau hyperphosphorylation, it may be driven by preaggregate Tau or highly localized bioactive Tau species in wake-/sleep-promoting neurons.

Wakefulness is controlled by discrete populations of wake-promoting neurons, the best studied of which are the orexinergic/hypocretin neurons of the lateral hypothalamus (LH), and the noradrenergic neurons of the LC (Scammell et al., 2017). In nocturnal species, including mice, these wake-promoting brain regions are more active in the dark phase. We speculate that in PS19 mice wake-promoting brain regions may be hyperactive during the dark phase, driving the hyperarousal phenotype. In accord with this idea, it was recently shown that sleep fragmentation occurring as a result of “natural aging” was caused by hyperexcitability of the orexin-LH neurons (Li et al., 2022). The LC is one of the first brain regions to exhibit Tau pathology in human AD patients or even seemingly healthy younger adults and also shows early-stage pathology in PS19 mice (Braak et al., 2011; Kelly et al., 2017; Zhu et al., 2018; Fig. 7). This suggests that LC may be one of the first brain regions affected in tauopathies and one of the most vulnerable brain regions to sleep loss. Moreover, LC neurons innervate vast territories of the forebrain, and the spread of pathological Tau variants across neuronal synapses has been proposed to be mediated by LC axons (Iba et al., 2015; Holth et al., 2019). It is debated whether Tau drives neuronal hypo- or hyperexcitability, but an emerging trend is that Tau may drive initial hyperexcitability in the early stages of the disease, which then segues into a progressive hypoexcitation as neurons begin to succumb to pathology (Crimins et al., 2012; Hall et al., 2015; Targa Dias Anastacio et al., 2022). We speculate that in PS19 mice, a preaggregate, bioactive form of Tau may drive hyperexcitability in wake-promoting LC neurons, driving the early-stage dark-phase hyperarousal phenotype we report. As hyperarousal progresses, loss of sleep and hyperactivation of LC neurons may initiate a feedforward cycle driving Tau pathology and neuron loss in the LC, leading to further changes in sleep behavior and promoting cognitive decline, consistent with prior reports (Chalermpalanupap et al., 2018; Zhu et al., 2018).

### Sleep changes and association with forebrain synapses

Pathological Tau is known to spread between neurons via synaptic connections. Importantly, Tau release from neurons is a function of the sleep–wake cycle and is exacerbated by sleep disruption and heightened neuronal activity (Holth et al., 2019). In healthy neurons, Tau is localized to axons but becomes mislocalized to the dendritic compartment and postsynapse during disease progression (Dejanovic et al., 2018; Ittner and Ittner, 2018). We speculated that Tau mislocalization to forebrain synapses may also be driven by sleep disruption. Counter to our prediction, we did not see a correlation between sleep measures and synaptic Tau pathology in the cortex. Moreover, acute 4 h of sleep deprivation or 30 d of chronic sleep disruption in 6-month-old PS19 mice did not result in any change in forebrain synapse–associated phospho-Tau compared with undisturbed controls. These findings suggest that sleep disruption may not directly cause Tau mistargeting to synapses. Consistent with these findings, and mentioned above, Holth et al. (2017) reported that sleep amount was negatively correlated with Tau pathology in sleep-promoting regions of the brainstem, but not in the cortex. One possibility is that our sleep manipulations were insufficient and that more severe sleep disruption treatments (longer total deprivation, or more sustained chronic disruption) may drive an increase in Tau burden at the synapse. Another possibility is that sleep disruption may indirectly promote Tau pathology in synapses; for example, sleep disruption is known to exacerbate neuroinflammation (C. Wang and Holtzman, 2019; Morrone et al., 2023). We suggest that while sleep disruption does not seem to be causally related to forebrain synapse Tau burden, sleep disruption may synergize with synapse pathology to contribute to cognitive decline and neuron loss. Further research on the interaction between Tau pathology and sleep loss in the forebrain is required to address this.

### Sex-specific difference in onset of, and response to, sleep disruption

We began our study by asking when sleep disruption and Tau pathology occur in PS19 mice in an age and sex-dependent manner. This design is in accordance with recent recommendations that experiments should include both sexes (Shansky, 2019), which has not been well reflected in past AD animal research studies. We noted numerous important sex differences throughout our study. First, PS19 females showed an earlier onset of hyperarousal (3 months) compared with males (6 months). In response to CSD treatment, females, but not males, show a hippocampal-specific adaptation involving an upregulation of AMPA and NMDA-type glutamate receptors and other synaptic proteins. Further, we show that CSD treatment was able to accelerate the onset of cognitive decline as measured using the Morris water maze in male PS19 mice, whereas females were found to be resilient. It is possible that female-specific upregulation of hippocampal synaptic proteins in response to CSD may form the basis of a type of “cognitive reserve” that was able to protect the cognition of female PS19 mice, whereas males, lacking this putative adaptive mechanism, may be more vulnerable to the negative consequences of sleep loss. We note that this exciting possibility is highly speculative, and considerably more work will be needed to fully support this idea. Of note, men have been shown to sleep more than women, as women experience unique sleep disruptions during child-rearing. Men, however, experience more sleep architecture disruptions that worsen with age (Ehlers and Kupfer, 1997; Redline et al., 2004; Mander et al., 2017). It can be hypothesized that females develop more adaptations for sleep loss, leading to the disparity seen in the exaggerated progressive decline in sleep in males (Swift et al., 2020). The current limited pool of AD therapies does not account for sex differences; however, our data provides robust evidence for the recognition of sex as a biological variable that deserves considerable attention.

## Conclusion

We show that sleep disruption, in the form of dark-phase hyperarousal, is an early symptom of disease progression in PS19 tauopathy model mice. Chronic sleep disruption accelerated the onset of cognitive decline in PS19 males. Females show an earlier onset of hyperarousal; however, females showed resilience to the effects of chronic sleep disruption. We further conclude that while sleep disruption is not a direct driver of Tau hyperphosphorylation in the forebrain synapses, sleep disruption likely interacts with synaptic Tau hyperphosphorylation to drive cognitive decline.

## Synthesis

Reviewing Editor: Christopher Colwell, UCLA School of Medicine Decisions are customarily a result of the Reviewing Editor and the peer reviewers coming together and discussing their recommendations until a consensus is reached. When revisions are invited, a fact-based synthesis statement explaining their decision and outlining what is needed to prepare a revision will be listed below. The following reviewer(s) agreed to reveal their identity: Sigrid Veasey, Seonil Kim.

At present, it is suspected but has not been established that sleep disruption can influence the progression of tauopathy. The authors address this unknown by examining sex, age, sleep and tau interactions in the P301S murine model of tauopathy, first characterizing the temporal progression of sleep/wake/motor behaviors in the two sexes across the lifespan of PS19 mice and relating these changes to synaptic soluble phosphorylated tau changes in cortical post-synaptosomes and then by examining the effects of acute and chronic sleep disruption on synaptic soluble phosphorylated tau changes in cortical and hippocampal post-synaptosomes and spatial memory. This is the first description of sleep-like behaviors with both sexes compared for this model. The work also directly tests the impact of acute and chronic sleep disruption on synaptic soluble phosphorylated tau changes in cortical and hippocampal post-synaptosomes. This is a comprehensive study that includes memory testing, sleep assessment, and assessment of post-synaptic tau phosphorylation and excitatory receptors in the cortex and hippocampus. The results extend previous work in important ways. There are a few concerns with the presentation of data and discussion as outlined below in a more detailed summary.

Sleep assessment/findings: Although electrographic signals are considered a gold standard for sleep studies, there are several strengths to using the piezo-electric system used in the present study. First, the electrodes may disrupt the blood brain barrier and or cause inflammation that may influence brain pathology. Second, as nicely illustrated here, the mice can be studied for many days to add robustness to the measure, and 7 days gets through the estrus cycle for females. The method has been validated, and there may be some overestimate of wake, given REM sleep behavior disorder in older mice, but REM sleep represents a small percentage of the total sleep. In males the data are consistent with sleep/wake changes previously described in males (Holth, et al., 2017). By adding the hourly data, an interesting change emerges in female mice which is a loss of the ultradian rhythm of sleep across the dark period, and this may be a clue for where to look for pathological changes. Thus, the current findings add to published work in this area. When parceled into NREM-like and REM-like, it appears that this ultradian rhythm was present for NREM sleep and this is lost over time in P301S mice, male and female. The authors should comment on this new finding.

Tau assessment/findings: The findings should be termed tau hyperphosphorylation rather than tau pathology, as this is soluble fraction p-tau. Multiple phosphorylation sites have been assessed which is a strength. Consistent differences are shown in raw data Fig. 3, with genotype and sex differences. The differences should be quantified (semi-quantified) as band density normalized to Hcs70 band density, as it appears that males have greater p-tau for several sites and total tau. Authors should state what band sizes were measured for each western. Additionally, because westerns were done, it would be nice to see kD bands for <50kD as there may be increased fragmentation in a particular group. The authors must acknowledge that they did not measure synaptic density and thus tau into synapses may have resulted n loss of synapses, but this would be missed in the current analyses. The Westerns are detecting soluble and miss insoluble tau that may be evident by immunohistology (this just needs to be mentioned not performed for this work).

Sleep disruption procedure/assessment of p-tau effects and synaptic proteins: Both protocols are well-established, no concerns. The variability is Fig. 4 C,E, is strikingly high. There are very large differences rostral-caudal, where highest p-tau is in the entorhinal cortex and then posterior hippo, and least is most rostral cortex. Were the same Bregma AP locations used for dissections across animals? It is indeed surprising to see such dramatically increased synaptic proteins in female mice exposed to chronic sleep disruption. It would be important to re-examine and add tubulin, actin, or homer1A or to measure males and females in the same gel (is this sleep disruption increase specific to excitatory receptor proteins or some overall effect on all synaptic proteins, or was this a protein loading error for the hippocampus (housekeeper suggests not the case, but run separately)?

In Fig. 6, since the variables are two; sleep disruptions and tau transgenes, two-way ANOVA analysis with the post-hoc test is more appropriate than one-way ANOVA test.

There are several typos (E.g. "casual link" in line 14).

In the method of postsynaptic density preparation, samples were subjected to ultra-centrifugation at "25" x g in line 150, 158 and 161. The authors should confirm if these are correct.

Perhaps, it would be easier to understand the results if figures 6, 7, and 8 were rearranged.
