# Supplementary figures and images for: Sleep Disruption Precedes Forebrain Synaptic Tau Burden and Contributes to Cognitive Decline in a Sex-Dependent Manner in the P301S Tau Transgenic Mouse Model
Source: eNeuro. 2024 Jun 14;11(6):ENEURO.0004-24.2024. doi: 10.1523/ENEURO.0004-24.2024 (PMC11209651; doi:10.1523/ENEURO.0004-24.2024)

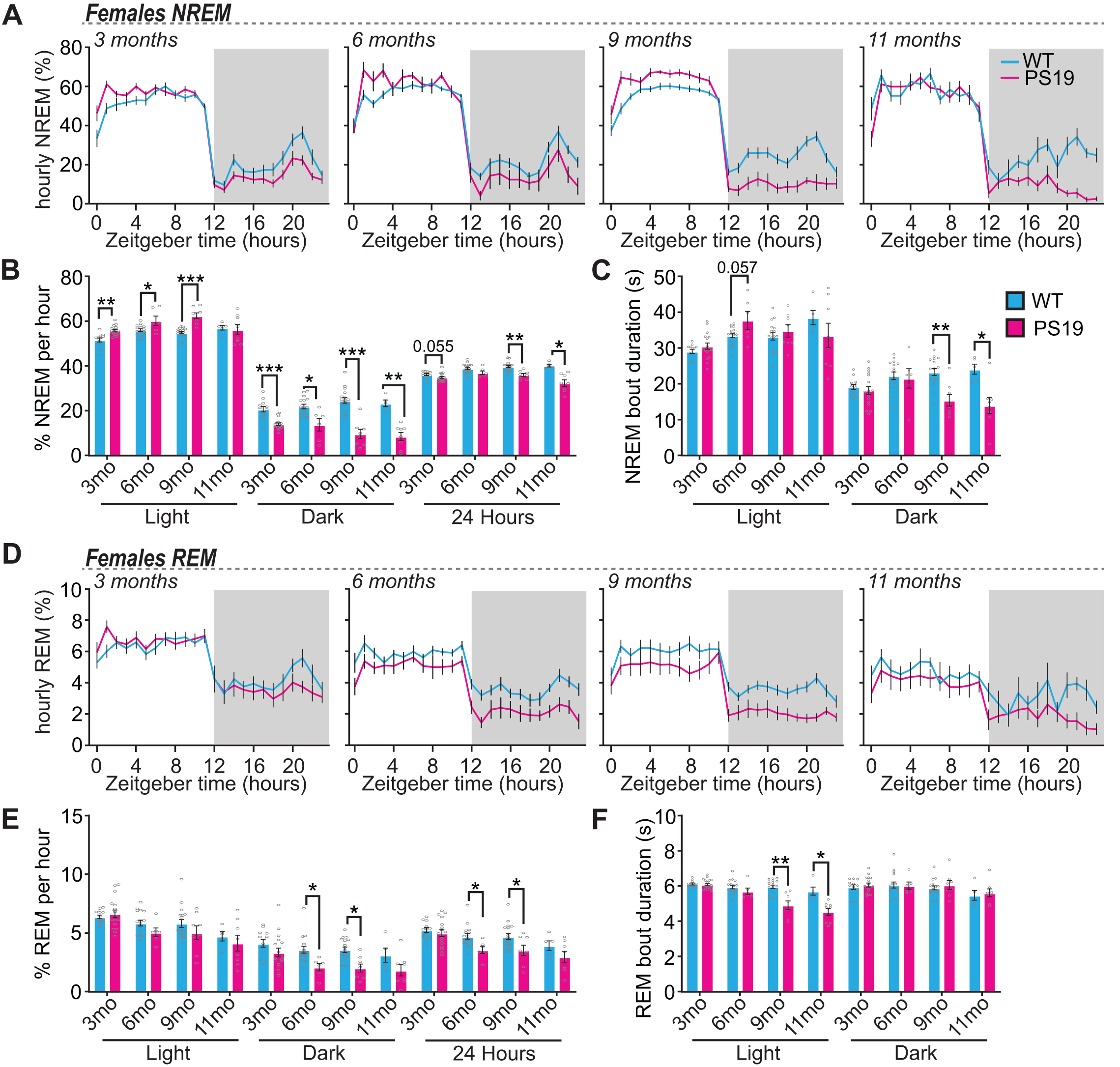

Supplement: Figure 1-1 — PS19 female mice exhibit progressive decrease in REM and NREM sleep. (A) 24hr trace of NREM sleep in female WT (blue line) PS19 (pink line) mice at 3, 6, 9, 11 months. Grey bars in sleep traces indicate dark phase. (B and C) Quantification of average hourly NREM sleep amount (B) and NREM sleep bout length in seconds (C). (D) 24hr trace of REM sleep in female WT (blue line) PS19 (pink line) mice at 3, 6, 9, 11 months. Grey bars in sleep traces indicate dark phase. (E and F) Quantification of average hourly REM sleep amount (E) and REM sleep bout length in seconds (E). Data separated into 12hrs of dark and light phases. N = 5-17/age/genotype. *p < 0.05, **p < 0.01, ***p < 0.001 Unpaired two-tailed student’s t-test. Error bars indicate ± SEM. Download Figure 1-1, TIF file. [file eneuro-11-ENEURO.0004-24.2024-s003.tif]

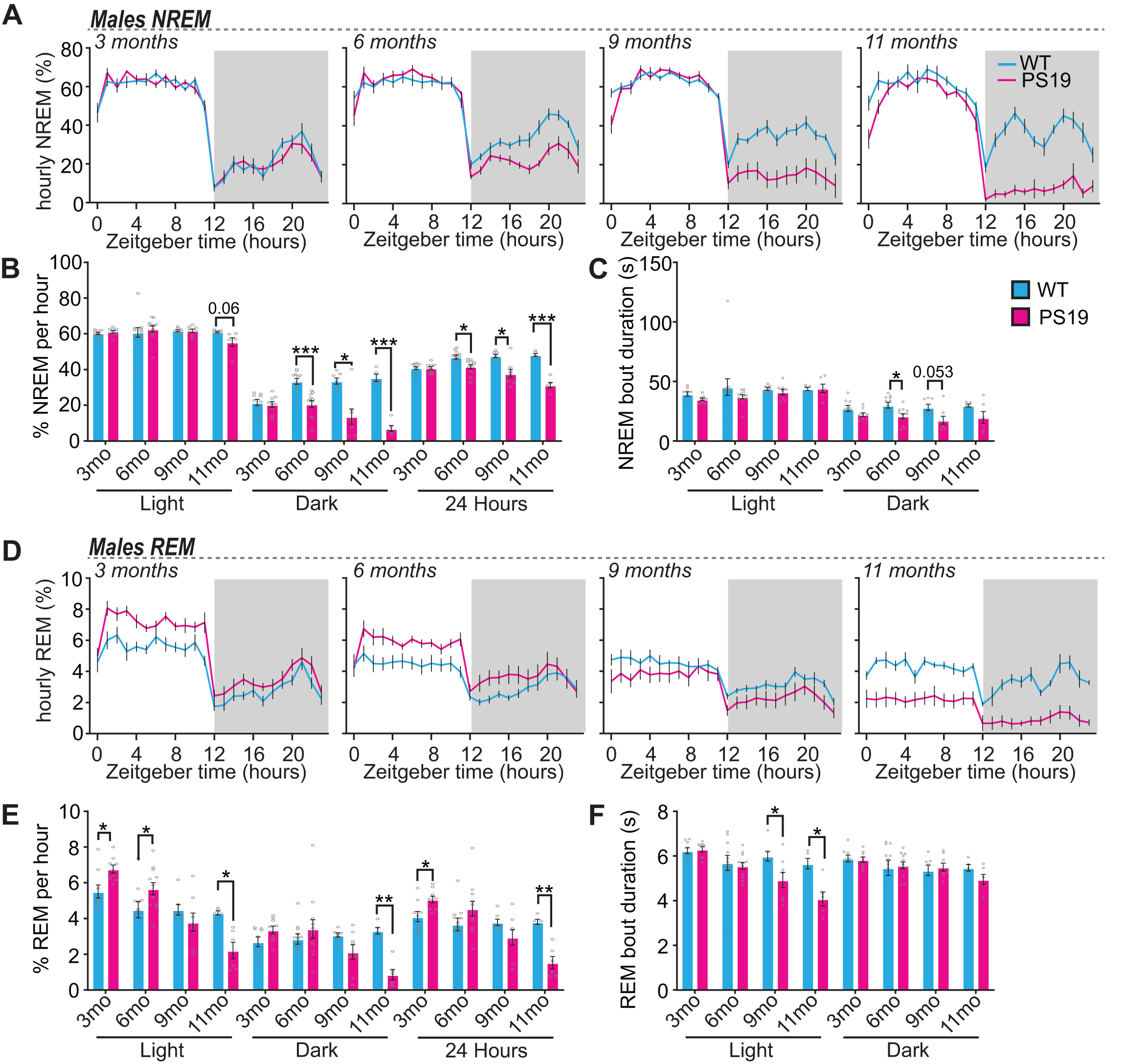

Supplement: Figure 1-2 — PS19 male mice exhibit progressive decrease in REM and NREM sleep. (A) 24hr trace of NREM sleep in male WT (blue line) PS19 (pink line) mice at 3, 6, 9, 11 months. Grey bars in sleep traces indicate dark phase. (B and C) Quantification of average hourly NREM sleep amount (B) and NREM sleep bout length in seconds (C). (D) 24hr trace of REM sleep in male WT (blue line) PS19 (pink line) mice at 3, 6, 9, 11 months. Grey bars in sleep traces indicate dark phase. (E and F) Quantification of average hourly REM sleep amount (E) and REM sleep bout length in seconds (E). Data separated into 12hrs of dark and light phases. N = 5-17/age/genotype. *p < 0.05, **p < 0.01, ***p < 0.001 Unpaired two-tailed student’s t-test. Error bars indicate ± SEM. Download Figure 1-2, TIF file. [file eneuro-11-ENEURO.0004-24.2024-s004.tif]

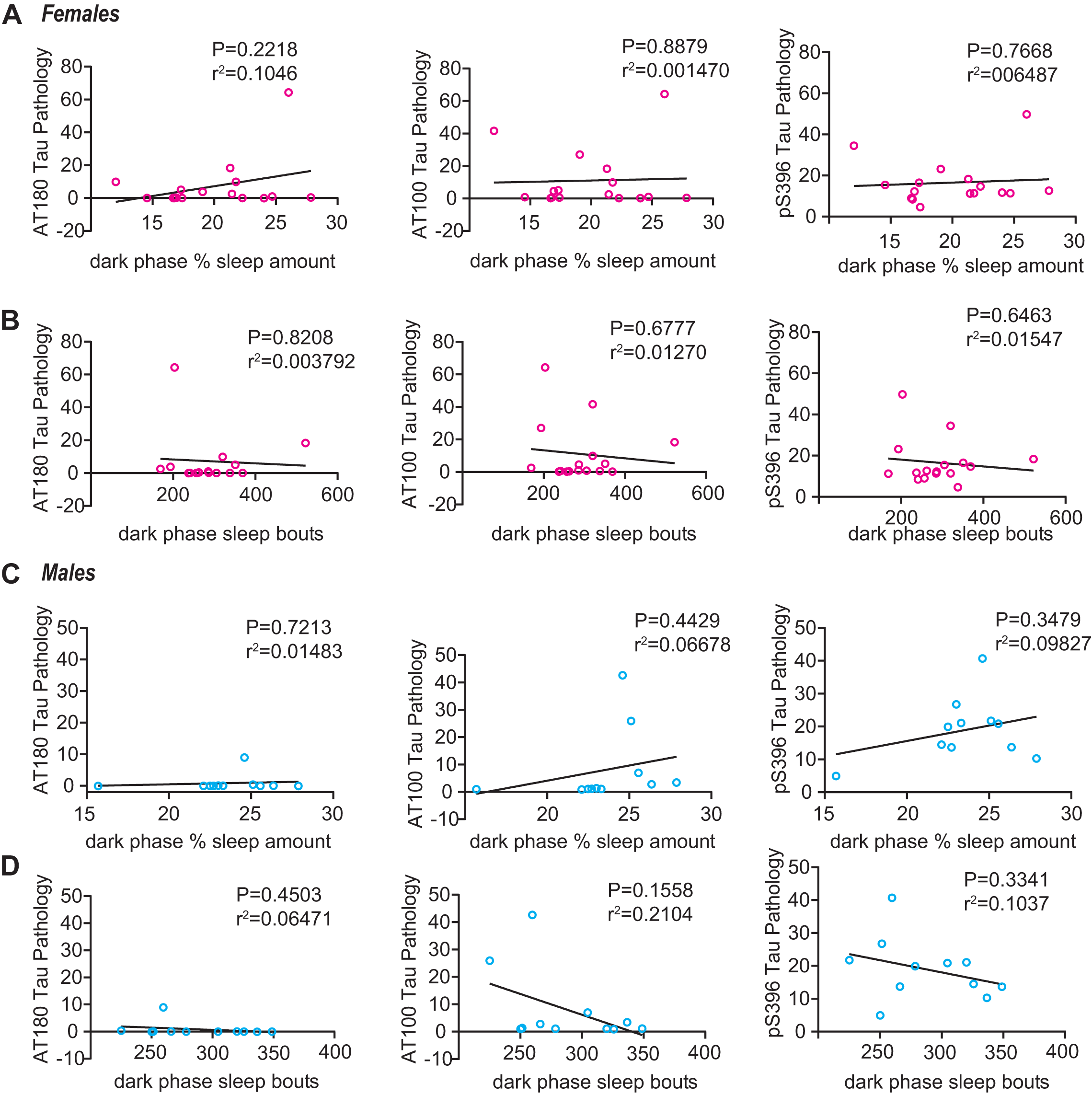

Supplement: Figure 4-1 — Decreased sleep amount in PS19 Tau tg mice is not predicative of AT8 Tau pathology in the cortex (continued). Western blots analysis of AT180, AT100, pS396 (see Figure 3) correlated to dark phase sleep measures in 6-month (early phase) PS19 females and males. (A and B) Correlation analysis of AT180, AT100, pS396 Tau pathology expression in the cortex of PS19 females with average dark phase hourly sleep (A) or sleep bout length in seconds (B). N = 16 PS19 females. (C and D) Correlation analysis of AT180, AT100, pS396 Tau pathology expression in the cortex of PS19 males with average dark phase hourly sleep (C) or sleep bout length in seconds (D). N = 11 PS19 males. All antibodies normalized to loading control. No significance (Pearson correlation). Download Figure 4-1, TIF file. [file eneuro-11-ENEURO.0004-24.2024-s002.tif]
